# Anti-aging effects of long-term space missions, estimated by heart rate variability

**DOI:** 10.1038/s41598-019-45387-6

**Published:** 2019-06-20

**Authors:** Kuniaki Otsuka, Germaine Cornelissen, Yutaka Kubo, Koichi Shibata, Koh Mizuno, Hiroshi Ohshima, Satoshi Furukawa, Chiaki Mukai

**Affiliations:** 1 Executive Medical Center, Totsuka Royal Clinic, Tokyo Women’s Medical University, Tokyo, Japan; 2 Halberg Chronobiology Center, University of Minnesota, Minneapolis, Minnesota USA; 3 Department of Medicine, Tokyo Women’s Medical University, Medical Center East, Tokyo, Japan; 4 Faculty of Education, Tohoku Fukushi University, Miyagi, Japan; 5 Space Biomedical Research Group, Japan Aerospace Exploration Agency, Tokyo, Japan

**Keywords:** Quality of life, Ageing

## Abstract

Reports that aging slows down in space prompted this investigation of anti-aging effects in humans by analyzing astronauts’ heart rate variability (HRV). Ambulatory 48-hour electrocardiograms from 7 astronauts (42.1 ± 6.8 years; 6 men) 20.6 ± 2.7 days (ISS01) and 138.6 ± 21.8 days (ISS02) after launch were divided into 24-hour spans of relative lower or higher magnetic disturbance, based on geomagnetic measures in Tromso, Norway. Magnetic disturbances were significantly higher on disturbed than on quiet days (ISS01: 72.01 ± 33.82 versus 33.96 ± 17.90 nT, P = 0.0307; ISS02: 71.06 ± 51.52 versus 32.53 ± 27.27 nT, P = 0.0308). SDNNIDX was increased on disturbed days (by 5.5% during ISS01, P = 0.0110), as were other HRV indices during ISS02 (SDANN, 12.5%, P = 0.0243; Triangular Index, 8.4%, P = 0.0469; and TF-component, 17.2%, P = 0.0054), suggesting the action of an anti-aging or longevity effect. The effect on TF was stronger during light (12:00–17:00) than during darkness (0:00–05:00) (P = 0.0268). The brain default mode network (DMN) was activated, gauged by increases in the LF-band (9.7%, P = 0.0730) and MF1-band (9.9%, P = 0.0281). Magnetic changes in the magnetosphere can affect and enhance HRV indices in space, involving an anti-aging or longevity effect, probably in association with the brain DMN, in a light-dependent manner and/or with help from the circadian clock.

## Introduction

Aging is the inevitable time-dependent decline in physiological organ function and a major risk factor for cardiovascular disease, cancer and Alzheimer’s disease. Novel treatments and translational approaches have been tried to prevent, delay, alleviate or even reverse age-related diseases. Maximal longevity, however, appears unchanged. Space research is bringing insight on how to directly intervene against the aging hallmarks and prevent age-related diseases. This possibility may exist because spaceflight has complex effects on the physiology of organisms, such as reduced gravity and different electromagnetic surroundings, which induce changes to almost every system in the body.

The Sun markedly affects most activities on Earth, including its solid, liquid and gas states. Whether life started on Earth or in the cosmos (panspermia hypothesis), it evolved following long physical/chemical processes, so that life on Earth matches what this planet requires^1,2^. Now, humans plan to live on the moon or Mars^3,4^. Spaceflight opens new opportunities to learn about capabilities of the human body that could not be studied on Earth. Spaceflight is known to dramatically alter cardiovascular dynamics^5–11^, also posing significant risks, such as an overall greater than 10-fold faster onset and time course of muscle and bone atrophy^12–15^. Microgravity is one of the crucial contributors to these observed physiological changes.

How microgravitational space environments affect aging is not well understood^16,17^. Honda *et al*.^18^ reported that spaceflight in *Caenorhabditis elegans* suppressed the formation of transgenically-expressed polyglutamine aggregates, which normally accumulate with increasing age. Inactivation of each of the seven genes that were down-regulated in space was found to extend lifespan on the ground. Aging in *Caenorhabditis elegans* seemed to be slowed through neuronal and endocrine responses to space environmental cues.

Whether similar anti-aging effects apply to astronauts during long-term missions in space is investigated herein by analyzing heart rate variability (HRV) on quiet versus magnetically-disturbed days. Exposure to weak geomagnetic fields is associated with biological effects^1,19–22^. Previously we showed that magnetic disturbances suppress HRV primarily in the very-low frequency region, which is clinically important since its reduction is a predictor of morbidity and mortality from cardiovascular disease^23,24^. HRV in the minutes to hours (very-low to ultra-low frequency) range is also a powerful predictor of longevity in clinically healthy people.

## Methods

### Experimental conditions on the ISS

Universal Time Coordinated (UTC) is used aboard the ISS. The windows are covered during night hours to give the impression of darkness because the station experiences 16 sunrises and sunsets per day.

A typical day for astronauts begins with awakening at 06:00, followed by post-sleep activities and a morning inspection of the station. The crew then eats breakfast and takes part in a daily planning conference with Mission Control before starting work at around 08:10. The first scheduled exercise of the day follows, after which the crew continues work until 13:05. Following a one-hour lunch break, the afternoon consists of more exercise and work before astronauts carry out their pre-sleep activities beginning at 19:30, including dinner and a crew conference. The scheduled sleep span begins at 21:30^25^.

### Subjects

Of the 10 healthy astronauts who participated in ISS JAXA investigation named “Biological Rhythms 48 Hrs”, 3 were excluded because their data were insufficient for the purpose of this study. There was partial shortage of their pre- and post-flight ECG data probably due to poor contact of the electrodes. The mean (±SD) age of the 7 healthy astronauts (6 men, 1 woman) included in this investigation was 42.1 ± 6.8 years. Their mean stay in space was 151.3 ± 21.8 days. Astronauts had passed class III physical examinations from the National Aeronautics and Space Administration (NASA). The study was approved by the Institutional Review Boards of NASA, ESA (European Space Agency) and JAXA (Japan Aerospace Exploration Agency). Informed consent was obtained from all subjects. A detailed explanation of the study protocol was given to the subjects before they gave written, informed consent, according to the Declaration of Helsinki Principles. All methods were performed in accordance with the JAXA/ESA/NASA guidelines and regulations.

### Experimental protocol

Ambulatory around-the-clock 48-hour electrocardiographic (ECG) records were obtained by using a two-channel Holter recorder (FM-180; Fukuda Denshi). Measurements were made four times: once before flight (Pre); two times during flight on the International Space Station (ISS): ISS01 (20.6 ± 2.7, 18–25 days), and ISS02 (138.6 ± 21.8, 101–159 days) after launch; and once after return to Earth (Post). The 48-hour records during ISS01 and ISS02 were divided into 24-hour spans of relative lower or higher magnetic disturbance for comparison of HRV endpoints between “quiet” and “disturbed” conditions.

### Assessment of space magnetics

The ISS is protected from the space environment by Earth’s magnetic field. The ISS orbits the Earth every 90 min at an altitude of 330 to 480 km. Izumi *et al*.^26^ reported that the geomagnetic strength at 400 km is about 4/5 or 3/4 that at sea level. Lacking direct measurement on the ISS, we used geomagnetic measurements at 1-min intervals from the Auroral Observatory of the University of Tromsø, in Tromsø, Norway (69°39′N, 18°56′E): total intensity (F in nT), declination (D, angle between geographic and magnetic north, in degrees), inclination (I, angle between horizontal plane and magnetic direction, in degrees), horizontal intensity (H in nT), and vertical intensity (Z in nT).

Historically, magnetic records obtained from the ground have widely been used to study physical processes that occur in the near-Earth environment. Kamide *et al*.^27^ proposed various models of the estimation of ionospheric electric field, ionospheric currents and field-aligned currents from ground magnetic records, along with incoherent scatter radars, satellite measurements of X-ray and UV aurorae. This type of study over the past two hundred years about the structure and temporal changes of the Sun–Earth space, now called space weather, has now become of interest to space science. However, it is still extremely difficult to specify the state of geomagnetics in space^2,28–31^. Humans are not consciously aware of changes in space weather. Except for the presence of aurorae, which occur when magnetic disturbances exceed those encountered in the current study, external cues are also lacking.

### Analysis of HRV

Data collection and measurement procedures were conducted as previously reported^8–11^. Briefly, for HRV measurements, QRS waveforms were read from continuous ECG records. The RR intervals between normal QRS waveforms were extracted as normal-to-normal (NN) intervals, which were A/D converted (125-Hz) with 8-ms time resolution. After the authors confirmed that all artifacts were actually removed and that the data excluded supraventricular or ventricular arrhythmia, time-domain HRV indices (SDNN, SDANN, SDNNIDX and Triangular Index, TI), and conventional frequency-domain measures (HF: 0.15–0.40 Hz, LF: 0.04–0.15 Hz, and VLF: 0.003–0.04 Hz)^32^ were obtained with the MemCalc/CHIRAM (Suwa Trust GMS, Tokyo, Japan) software^33^. Time series of NN intervals were also processed consecutively in 180-min intervals, progressively displaced by 5 min, to estimate TF (0.0001–0.50 Hz), ULF (0.0001–0.003 Hz), and the 1/f^β^-type scaling in HRV. Focus was placed on the frequency range of 0.0001–0.01 Hz (periods of 2.8 hours to 1.6 minutes).

Time series of NN intervals covering 5-min intervals were processed consecutively, and spectral power in four frequency regions were computed using the Maximum Entropy Method (MEM): LF-band (0.01–0.05 Hz), MF1-band (0.05–0.10 Hz), MF2-band (0.10–0.15 Hz), and HF-band (0.15–0.20 Hz) according to Baria *et al*.^11,34^. A positive response in these bands is hypothesized to indicate how astronauts adapt to the space environment: the LF- and MF1-bands reflect an activation of the DMN’s medial prefrontal cortex (mPFC), posterior parietal cortex, posterior portion of precuneus and posterior cingulate cortex (PCC), while the MF2- and HF-bands show an activation of the orbitofrontal and temporal cortex parts of the DMN^34^.

### Circadian differences in HRV response to magnetic disturbance in space

Circadian stage-dependent HRV responses to magnetic disturbances in space were assessed by subdividing the 24-hour day into 4 time spans of 5 hours each: morning (around 08:30, 06:00–11:00); daytime (around 14:30, 12:00–17:00); evening (around 20:30, 18:00–23:00); and night (around 02:30, 00:00–05:00).

### Statistical analyses

Data were expressed as mean ± standard deviation (SD). HRV endpoints were compared among the 4 sessions by 1-way ANOVA, with contrasts. Of particular interest were comparisons of ISS01 vs. Pre and ISS02 vs. Pre, which reflect the extent of astronauts’ adaptation to the space environment.

Comparisons between magnetically quiet and disturbed days used the two-sided paired-t test. Effects of magnetic activity on HRV endpoints were tested in 4 spans (morning, daytime, evening and night) for each astronaut by Student t-test, adjusted for unequal variances and unequal sample sizes according to Welch’s correction. Subsequently, the number of statistically significant individual responses during morning, daytime, or evening versus night was compared by χ^2^ or Fisher exact test.

The Stat Flex (Ver. 6) software (Artec Co., Ltd., Osaka, Japan) was used. P-values less than 0.05 were considered to indicate statistical significance.

## Results

### Assessment of space magnetics on the ISS

Geomagnetic indices from the Auroral Observatory of the University of Tromso are compared between quiet and disturbed days in Table 1. The 24-hour SD of Z (magnetic field’s vertical component) was about twice as large on disturbed than on quiet days (ISS01: 72.01 ± 33.82 versus 33.96 ± 17.90 nT, P = 0.0307; ISS02: 71.06 ± 51.52 versus 32.53 ± 27.27 nT, P = 0.0308), and so was the 24-hour SD of the declination. Variations in H (magnetic field’s horizontal component) and inclination were larger on disturbed than on quiet days during ISS01, while during ISS02 those in total intensity were larger. No differences were found in the 24-hour averages (Table 1).

**Table 1:** Comparison of 24-hour average, standard deviation (SD), minimum (Min) and maximum (Max) of Tromso’s magnetic indices between magnetically quiet and disturbed days during ISS01 and ISS02.

	Geomagnetic indices (units)	ISS01 (n = 7)						ISS02 (n = 7)					
		Quiet day		Disturbed day		Paired t test		Quiet day		Disturbed day		Paired t test	
		Mean	SD	Mean	SD	t	p	Mean	SD	Mean	SD	t	p
24-hour average	Decl (°)	6.00	0.33	6.04	0.35	1.512	0.1813	6.11	0.32	6.17	0.30	1.774	0.1265
	H (nT)	10893.8	13.6	10859.9	50.5	−1.789	0.1238	10839.3	93.9	10845.0	73.4	0.142	0.8917
	V (nT)	52358.0	50.0	52371.3	62.1	1.355	0.2243	52367.7	41.1	52380.0	38.1	1.177	0.2837
	Incl (°)	78.25	0.02	78.28	0.06	1.768	0.1275	78.31	0.10	78.30	0.08	−0.140	0.8934
	Total (nT)	53479.4	46.7	53485.7	55.9	0.966	0.3712	53477.9	42.7	53491.2	35.3	0.994	0.3588
SD	Decl (°)	0.15	0.06	0.32	0.12	**5**.**799**	**0**.**0012**	0.14	0.11	0.32	0.26	**2**.**574**	**0**.**0421**
	H (nT)	70.36	39.46	135.26	40.85	**7**.**058**	**0**.**0004**	77.43	71.32	115.07	85.37	1.337	0.2298
	V (nT)	33.96	17.90	72.01	33.82	**2**.**812**	**0**.**0307**	32.53	27.27	71.06	51.52	**2**.**809**	**0**.**0308**
	Incl (°)	0.08	0.04	0.15	0.05	**6**.**181**	**0**.**0008**	0.08	0.07	0.13	0.10	1.598	0.1612
	Total (nT)	35.67	15.49	59.89	25.39	***2***.***323***	***0***.***0592***	35.47	29.68	67.23	43.72	**2**.**477**	**0**.**0480**
Min	Decl (°)	5.59	0.29	5.17	0.49	***−1***.***973***	***0***.***0959***	5.60	0.38	5.46	1.05	−0.441	0.6749
	H (nT)	10660.2	132.5	10355.5	299.7	**−3**.**315**	**0**.**0161**	10538.5	315.6	10411.8	396.1	−1.113	0.3085
	V (nT)	52265.3	69.1	52165.2	118.1	***−1***.***970***	***0***.***0964***	52224.4	125.3	52131.8	191.4	−1.450	0.1971
	Incl (°)	78.08	0.10	78.06	0.12	−0.288	0.7834	78.11	0.12	78.08	0.10	−0.625	0.5550
	Total (nT)	53369.9	88.7	53285.4	126.6	−1.631	0.1540	53323.5	141.5	53207.4	228.1	−1.399	0.2113
Max	Decl (°)	6.48	0.41	7.56	0.98	**3**.**329**	**0**.**0158**	6.94	0.91	8.30	2.05	**2**.**611**	**0**.**0400**
	H (nT)	11053.3	87.0	11062.7	104.8	0.164	0.8750	11008.9	103.5	11043.0	78.8	0.712	0.5033
	V (nT)	52471.1	80.8	52699.8	225.0	**2**.**721**	**0**.**0346**	52479.0	103.4	52669.9	252.3	**3**.**187**	**0**.**0189**
	Incl (°)	78.51	0.15	78.85	0.34	**3**.**245**	**0**.**0176**	78.63	0.34	78.77	0.43	1.217	0.2692
	Total (nT)	53579.4	64.2	53744.3	184.7	**2**.**602**	**0**.**0406**	53564.9	70.9	53737.3	202.1	**3**.**122**	**0**.**0205**

Decl (declination, angle between geographic and magnetic north, in degrees), H (horizontal intensity, in nT), V (vertical intensity, in nT), Incl (inclination, angle between horizontal plane and magnetic direction, in degrees), and Total (total intensity, in nT). SD, Min, and Max computed over 24-hour span.

When H_0_ (equality of means between quiet and disturbed days) is rejected at α = 0.05 (or 0.10), results of test are shown in bold-face (or italicized bold-face).

### Effects of magnetics on astronauts’ HRV in space

Alterations of HRV associated with geomagnetic activity are summarized in Table 2. A 4.0% decrease in the 24-hour average of HR (P = 0.0392) was observed during ISS01. Parasympathetic activity estimated by r-MSSD and pNN50 showed no statistically significant changes, whereas sympathetic activity estimated by LF/HF increased by 13.7% during ISS02 (Table 2, top). No difference in β was found, suggesting that magnetic disturbances of magnitude similar to that taking place during ISS01 and ISS02 may not influence the intrinsic cardiovascular autonomic regulatory system.

**Table 2:** Effect of magnetic disturbance in space on various kinds of heart rate variability (HRV) in astronauts during ISS01 and ISS02.

		Unit/Frequency range (Hz)	ISS01 (n = 7) (20.6 days on ISS)					
			Quiet conditions		Disturbed conditions		Paired t-test	
			Mean	SD	Mean (%)	SD	t	p
HRV 1 (Indices of cardiovascular autonomic nervous activity)	β	0.0001–0.01	−0.8489	0.0939	−0.8148	0.0819	1.506	0.1828
	Heart Rate	bpm	74.1	7.8	71.9 (96.0%)	9.3	**−2**.**628**	**0**.**0392**
	NN-interval	msec	852.0	101.0	872.4 (97.1%)	111.9	***2***.***311***	***0***.***0602***
	r-MSSD	msec	29.7	14.5	31.1	15.1	1.854	0.1132
	pNN50	%	10.1	12.4	11.4 (113.3%)	13.1	***2***.***250***	***0***.***0654***
	LF/HF	(−)	5.75	1.61	5.92	2.17	0.629	0.5523
HRV 2 (Indices suggestive of anti-aging or longevity)	SDNN	msec	179.8	48.0	168.9	36.8	−0.827	0.4396
	SDANN	msec	156.8	43.4	150.1	32.2	−0.502	0.6333
	SDNNIDX	msec	62.9	18.2	66.3 (105.5%)	18.1	**3**.**629**	**0**.**0110**
	TI	(−)	41.6	11.8	38.6	8.1	−1.029	0.3430
	TF-c	0.0001–0.5	6173.0	3572.5	6748.4	3200.2	1.048	0.3350
HRV 3 (Frequency-domain indices)	ULF-c	0.0001–0.003	2881.2	1697.8	3067.7	1385.3	0.409	0.6967
	VLF-c	0.003–0.04	2114.0	1160.7	2412.8 (114.1%)	1195.7	**4**.**718**	**0**.**0033**
	LF-c	0.04–0.15	851.4	439.5	930.7	518.5	1.139	0.2981
	HF-c	0.15–0.40	271.8	355.2	283.2	341.1	1.215	0.2701
HRV 4 (Indices related to brain’s DMN activity)	LF-band	0.01–0.05	1361.4	728.6	1369.6	678.9	0.043	0.9671
	MF1-band	0.05–0.10	518.2	238.7	856.9	879.0	1.196	0.2770
	MF2-band	0.10–0.15	190.8	147.8	210.7	173.5	1.333	0.2311
	HF-band	0.15–0.20	116.2	184.1	106.5	139.2	−0.512	0.6271
		**Unit/Frequency range (Hz)**	**ISS02 (n** = **7) (138**.**6 days on ISS)**					
			**Quiet conditions**		**Disturbed conditions**		**Paired t-test**	
			**Mean**	**SD**	**Mean (%)**	**SD**	**t**	**p**
HRV 1 (Indices of cardiovascular autonomic nervous activity)	β	0.0001–0.01	−0.8697	0.0774	−0.8965	0.0518	−0.932	0.3873
	Heart Rate	bpm	74.6	8.2	74.6	6.5	0.029	0.9775
	NN-interval	msec	843.6	100.7	840.4	83.2	−0.310	0.7671
	r-MSSD	msec	30.9	15.6	30.3	12.9	−0.491	0.6407
	pNN50	%	10.9	12.6	10.1	10.3	−0.787	0.4612
	LF/HF	(−)	6.24	2.89	7.10 (113.7%)	3.43	**3**.**219**	**0**.**0182**
HRV 2 (Indices suggestive of anti-aging or longevity)	SDNN	msec	166.1	48.8	182.8 (110.0%)	62.4	***2***.***099***	***0***.***0806***
	SDANN	msec	144.5	41.7	162.6 (112.5%)	54.0	**2**.**989**	**0**.**0243**
	SDNNIDX	msec	63.0	22.7	67.1 (106.5%)	21.0	**2**.**602**	**0**.**0405**
	TI	(−)	35.7	11.6	38.7 (108.4%)	10.7	**2**.**494**	**0**.**0469**
	TF-c	0.0001–0.5	6520.1	3326.2	7640.8 (117.2%)	3663.1	**4**.**242**	**0**.**0054**
HRV 3 (Frequency-domain indices)	ULF-c	0.0001–0.003	2617.0	1055.0	3427.2 (131.0%)	1544.7	**3**.**422**	**0**.**0141**
	VLF-c	0.003–0.04	2499.8	1437.5	2816.1 (112.7%)	1444.8	**2**.**779**	**0**.**0320**
	LF-c	0.04–0.15	1017.3	649.4	1108.5 (109.0%)	658.7	***2***.***164***	***0***.***0737***
	HF-c	0.15–0.40	284.4	354.6	262.5	312.0	−1.181	0.2825
HRV 4 (Indices related to brain’s DMN activity)	LF-band	0.01–0.05	1617.6	894.5	1773.9 (109.7%)	854.8	***2***.***171***	***0***.***0730***
	MF1-band	0.05–0.10	618.8	402.0	680.3 (109.9%)	414.8	**2**.**878**	**0**.**0281**
	MF2-band	0.10–0.15	240.4	184.3	250.8	168.4	0.663	0.5317
	HF-band	0.15–0.20	119.9	168.6	115.2	158.0	−0.820	0.4437

TI: Triangular Index; “-c” in TF-c, ULF-c, VLF-c, LF-c and HF-c stands for “-component”.

When H_0_ (equality of means between quiet and disturbed days) is rejected at α = 0.05 (or 0.10), results of test are shown in bold-face (or italicized bold-face).

### Heart rate variability related to possible anti-aging or longevity

**Figure 1:**
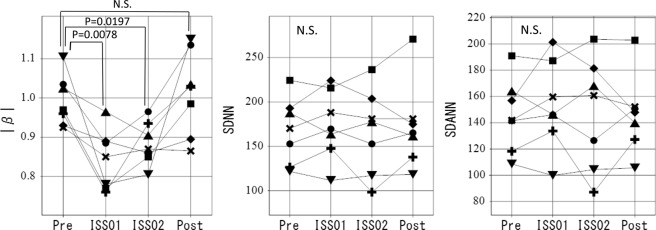
HRV plasticity observed in SDNN and SDANN, as indices of anti-aging or longevity. Although the cardiovascular autonomic regulatory function, reflected by β, was statistically significantly suppressed (left), SDNN (middle) and SDANN (right), computed over 24 hours, show a quality of HRV plasticity after about 6 months in space. Symbols were assigned to individual astronauts.

HRV indices suggestive of anti-aging or longevity effects include SDNN and SDANN. Although the cardiovascular autonomic regulatory function, reflected by β, was statistically significantly suppressed (Fig. 1, left), SDNN (Fig. 1, middle) and SDANN (Fig. 1, right), computed over 24 hours, show HRV plasticity already after about 3 weeks as well as after 6 months in space.

SDNNIDX is statistically significantly higher on disturbed than on quiet days during ISS01 (by 5.5%, P = 0.0110) and ISS02 (by 6.5%, P = 0.0405). During ISS02, 3 other HRV indices are elevated on disturbed days: SDANN (12.5%, P = 0.0243), Triangular Index (8.4%, P = 0.0469) and TF (17.2%, P = 0.0054) (Table 2, upper middle); SDNN shows a similar trend (10.0%, P = 0.0806).

**Figure 2:**
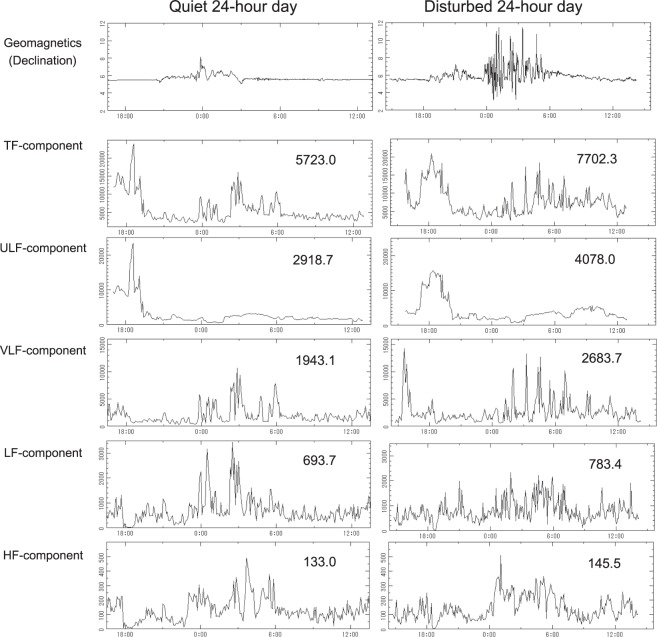
An astronaut’s circadian profiles of frequency-domain measures of HRV during ISS02 are compared between the quiet (left) and magnetically disturbed (right) day. HRV endpoints were computed by MEM spectra over 5-min or 180-min intervals and compared between the 2 days by Student’s t test. Values shown in individual graphs indicate spectral power cumulated over 24 hours. The larger values during disturbed vs. quiet conditions indicate a statistically significant response to magnetic disturbance in space. Not only TF (P < 0.0001), ULF (P = 0.0002) and VLF (P < 0.0001), but also LF (P = 0.0284) power (in msec^2^) were found to be statistically significantly higher on disturbed than on quiet days.

The larger TF power on disturbed days (ISS02) was contributed by higher ULF (31.0%, P = 0.0141) and VLF (12.7%, P = 0.0320) power; changes in LF (9.0%, P = 0.0737) and HF (−7.7%, P = 0.2825) power are not significant. SDNNIDX is known as a surrogate for VLF, which was already higher on disturbed days during ISS01 (14.1%, P = 0.0033) (Table 2, lower middle). The frequency-domain HRV endpoints of a 2-day record from one astronaut, split between the quiet and disturbed day, are illustrated in Fig. 2 together with the magnetic declination index for those two days. In this case, not only TF (P < 0.0001), ULF-(P = 0.0002) and VLF (P < 0.0001), but also LF (P = 0.0284) power is higher on the disturbed day, as shown by Student’s t-test.

### Involvement of the default mode network (DMN) in humans’ magnetoreception in space

**Figure 3:**
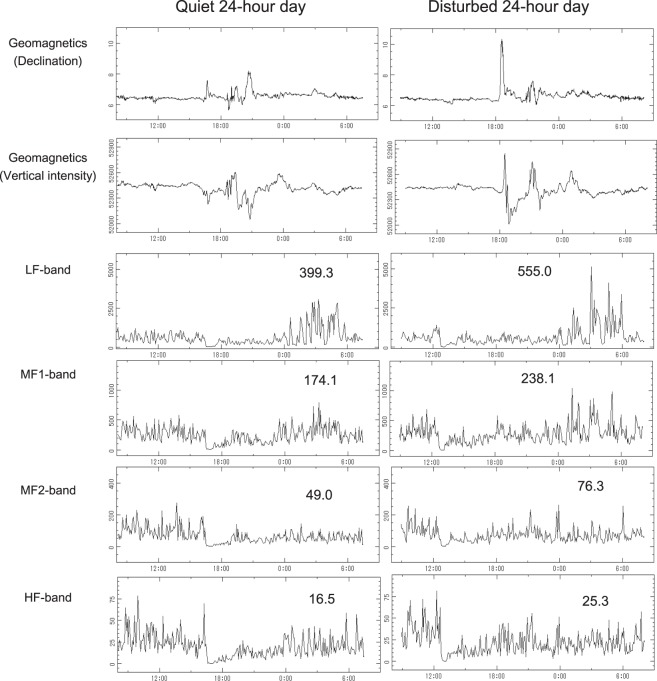
An astronaut’s circadian profiles of HRV measures related to DMN activity during ISS02 are compared between the quiet (left) and magnetically disturbed (right) day. HRV endpoints were computed by MEM spectra over 5-min or 180-min intervals and compared between the 2 days by Student’s t test. Values shown in individual graphs indicate spectral power cumulated over 24 hours. Spectral power (in msec^2^) of all 4 HRV bands (LF, MF1, MF2, and HF) were found to be higher on the magnetically disturbed than on the quiet day (P < 0.0001).

Examining the 4 HRV bands reflecting the brain’s DMN activity, MF1-band is statistically significantly higher on disturbed than on quiet days during ISS02 (9.9%, P = 0.0281). LF-band also shows a similar trend (9.7%, P = 0.0730) (Table 2, bottom). No differences in MF2- and HF-bands are found, however. Figure 3 illustrates the simultaneous records of the geomagnetic declination and vertical intensity together with HRV’s LF-, MF1-, MF2- and HF-bands of one astronaut. In this case, all 4 HRV bands reflecting the brain’s DMN activity are higher on the disturbed than on the quiet day (P < 0.0001), as shown by Student’s t-test.

### Circadian stage-dependent HRV response to magnetic disturbance in space

When subdividing the 24-hour day into 4 time spans of 5 hours each, centered around 08:30 (morning), 14:30 (daytime), 20:30 (evening) and 02:30 (night), the effect of magnetic activity in space was found to be circadian stage-dependent during ISS02. More astronauts had higher TF power on disturbed than on quiet days during the daytime and evening than during the night (P = 0.0308 by χ^2^ test). During ISS01, more astronauts also tended to have a higher VLF power and higher SDNNIDX on disturbed days during the daytime than during the night (P = 0.0943 by χ^2^ test), but they had less HF power in the evening than at night (P = 0.0962 by Fisher exact test) (Table 3).

**Table 3:** Circadian differences in each HRV response to magnetic disturbance in space: HRV change on disturbed vs quiet day (N of astronauts).

	ISS01					ISS02				
	Morning: HRV Increase?		Night: HRV Increase?		χ^2^ test	Morning: HRV Increase?		Night: HRV Increase?		χ^2^ test
	Yes	No	Yes	No	p	Yes	No	Yes	No	p
TF-c	2	5	3	4	0.5770	4	3	1	6	***0***.***0943***
ULF-c	2	5	2	5	1.0000	4	3	4	3	1.0000
VLF-c	2	5	3	4	0.5770	3	4	1	6	0.2367
LF-c	3	4	1	6	0.2367	2	5	2	5	1.0000
HF-c	3	4	3	4	1.0000	1	6	2	5	0.5148
SDNNIDX	2	5	1	6	0.5148	3	4	1	6	0.2367
	**Daytime: HRV Increase?**		**Night: HRV Increase?**		**χ** ^**2**^ **test**	**Daytime: HRV Increase?**		**Night: HRV Increase?**		**χ** ^**2**^ **test**
	**Yes**	**No**	**Yes**	**No**	**p**	**Yes**	**No**	**Yes**	**No**	**p**
TF-c	2	5	3	4	0.5770	5	2	1	6	**0**.**0308**
ULF-c	2	5	2	5	1.0000	4	3	4	3	1.0000
VLF-c	6	1	3	4	***0***.***0943***	2	5	1	6	0.5148
LF-c	4	3	1	6	0.2367	2	5	2	5	1.0000
HF-c	5	2	3	4	0.2801	1	6	2	5	0.5148
SDNNIDX	4	3	1	6	***0***.***0943***	1	6	1	6	1.0000
	**Evening: HRV Increase?**		**Night: HRV Increase?**		**χ** ^**2**^ **test**	**Evening: HRV Increase?**		**Night: HRV Increase?**		**χ** ^**2**^ **test**
	**Yes**	**No**	**Yes**	**No**	**p**	**Yes**	**No**	**Yes**	**No**	**p**
TF-c	3	4	3	4	0.5647	5	2	1	6	**0**.**0308**
ULF-c	3	4	2	5	0.5488	3	4	4	3	0.5930
VLF-c	1	6	3	4	0.3480	3	4	1	6	0.2367
LF-c	0	7	1	6	0.5000	2	5	2	5	1.0000
HF-c	0	7	3	4	***0***.***0962***	2	5	2	5	1.0000
SDNNIDX	0	7	1	6	0.5000	3	4	1	6	0.2367

“-c” stands for “-component”; P-values not corrected for multiple testing. Fisher’s exact test used instead of the χ^2^ test when comparison included a cell assuming a value of zero.

Note that similar results are obtained for VLF-c and SDNNIDX, considered a surrogate for VLF-c.

When H_0_ testing the equality of means between active (morning, daytime, or evening) and rest (night) spans is rejected at α = 0.05 (or 0.10), results of test are shown in bold-face (or italicized bold-face).

## Discussion

To examine whether the space environment may convey anti-aging effects in humans, HRV indices of astronauts on long-term missions were compared between days with relative lower or higher magnetic activity. Tromso’s geomagnetic measures served as surrogate since the ISS is protected by Earth’s magnetosphere. The fact that already during ISS01 SDNNIDX, a surrogate of the VLF-component, is increased on disturbed days suggests an anti-aging effect, as do similar increases during ISS02 in SDNN (a surrogate for the TF-component), SDANN, the Triangular Index and TF-component. The effect of magnetic activity on TF, suggesting an anti-aging effect, was found to be circadian stage-dependent, being more effective during light (12:00–17:00) than during dark (0:00–05:00) (P = 0.0268). The magnetic field in space also induced an activation of the brain’s DMN, gauged by HRV (increase in LF- and MF1-bands). An anti-aging effect in space may thus be mediated by the DMN in a light-dependent manner or/and with help from the circadian clock.

An anti-aging effect of the space environment was reported by Honda *et al*.^18^ on *Caenorhabditis elegans*. While their results indicate that space-flown worms age more slowly compared with ground control worms, the authors note that male Drosophila lived shorter on the ground after spaceflight compared to controls maintained on the ground through their lifetimes^18^. *Caenorhabditis elegans* is a classic animal model used to help understand human health concerns due to its short lifespan and ease of culture^35,36^. Simulated microgravity reportedly could affect early embryogenesis, reproduction and locomotion behavior, and potentially cause the oxidative stress and DNA damage in nematodes^37–41^.

In the study by Honda *et al*.^18^, the inactivation of each of seven genes that were down-regulated in space extended lifespan on the ground. These genes encode proteins that are likely related to neuronal or endocrine signaling: acetylcholine receptor, acetylcholine transporter, choline acetyltransferase, rhodopsin-like receptor, glutamate-gated chloride channel, shaker family of potassium channel, and insulin-like peptide. One of the genes identified encodes insulin, which is associated with metabolic control. In humans, insulin is also associated with modulation of lifespan. Li *et al*.^42^ showed that simulated microgravity could significantly increase the expression of p38 MAPK signaling in the intestine, which may mediate a protection mechanism for animals against the adverse effects from simulated microgravity. Studies in space may provide information about human life that cannot be learned on Earth, including lessons about aging.

The recently published NASA Twins Study describing a multidimensional analysis of a year-long human spaceflight^43^ also reported that telomeres predominantly lengthened on the ISS, suggesting an anti-aging effect of the space environment. This intriguing change at the chromosomal level, however, could also be interpreted as indicating an increased cancer risk, related to the enhanced exposure to ionizing radiation in space, warranting further investigation of how space weather may affect long-term missions in space.

HRV is defined as the beat-to-beat alterations to the sinus rhythm which result from the interactions between sympathetic and parasympathetic activity^32^. But HRV is probably more than an indicator of probable disturbances in the autonomous system^44^. HRV has been considered as a surrogate parameter on complex interactions within biological systems. The relation between reduced HRV and mortality risk was first shown by Wolf *et al*. in 1978^45^. High HRV is usually associated with good health, whereas low HRV might signify pathological changes, in agreement with the relation between lower HRV parameters and adverse cardiovascular outcomes^46–48^. HRV has recently been considered to reflect the state of the brain as much as the state of the heart^49–52^, and is viewed to also reflect the state of health and well-being^53–56^, a feature increasingly recognized as a hallmark of anti-aging. Whereas reduced HRV is associated with major risk factors of cognitive impairment, increased HRV indices (including SDNN, SDANN, Triangular Index and TF-, ULF- and VLF-components) are associated with better executive function in the middle aged and elderly^57–60^.

The atmosphere gradually ends a hundred kilometers above Earth’s surface. The Earth resides in a vast cavity called the magnetosphere, created by the interaction between Earth’s magnetic field and the solar wind, a gas of charged particles flowing continuously from the Sun. The ISS, which orbits the Earth at an altitude of 330 to 480 km, is also protected from the space environment by Earth’s magnetic field^2,26^. While conditions in the magnetosphere and on the Sun can potentially influence human health^1,2,19^, very little is known about their role in the adaptive response of humans to the space environment. The present study is the first to investigate this question.

Time- and frequency-domain HRV endpoints have served as potential markers of stress in organismic functions associated with adaptability and health. For instance, several recent reviews report significantly longer overall survival in cancer patients with higher versus lower HRV^55,61–63^. SDNNIDX and the VLF-component both increased in space in response to magnetic activity after just about one month on the ISS, meaning an activation of HRV in the 0.003–0.04 Hz frequency range. Definite increases in VLF, SDANN, TI, TF, and ULF were later observed on disturbed days during ISS02. SDNN also showed an increasing trend during ISS02. Exposure to variable magnetic fields in space resulted in an increase in several HRV endpoints, suggesting that aging may also be slowed down in astronauts in space, as it was for *Caenorhabditis elegans*^18^. The fact that VLF showed a strong response may point to anti-aging effects since VLF power is an indicator of health, lower VLF power being associated with arrhythmic death^46^ and inflammation^64,65^.

Why does the VLF-component act most prominently on the autonomous system? Historically, the physiological mechanisms involved in the long-term regulation mechanisms and autonomic nervous activity were linked to thermoregulation, the renin-angiotensin system and other hormonal factors^66,67^. Studying the autonomic nerves’ re-innervation in the transplanted heart, however, Kember *et al*.^68,69^ suggested that the VLF component was generated by the stimulation of afferent sensory neurons in the heart, which in turn activated various levels of the feedback and feed-forward loops in the heart’s intrinsic cardiac nervous system^70^. The VLF rhythm is now understood to be intrinsically generated by the heart itself, and that the amplitude and frequency of these oscillations are modulated by efferent sympathetic activity. As such, it should be fundamental to health and well-being. An enhanced VLF appearing soon after arrival at the ISS, persisting throughout the space mission may thus represent the most fundamental response to maintain health in space.

During the 5-year experimental span in solar cycle 24, an increased response of HRV was observed in space: 10% in SDNN, 12.5% in SDANN, 8.4% in TI, 17.2% in TF, 31.0% in ULF, 12.7% in VLF, and 9.0% in LF power (Table 2). By contrast, our previous 1998–2000 studies (during solar cycle 23) in a subarctic area indicated that magnetic storms, which involved larger magnetic disturbances than those observed during ISS01 and ISS02, suppressed HRV indices in 19 clinically healthy subjects^24^. The decrease in HRV was statistically validated for TF (−18.6%, P = 0.00009), primarily contributed to by VLF (−21.9%, P < 0.000001) in conjunction with ULF (−15.5%, P = 0.00865) and LF (−14.2%, P = 0.00187). Solar activity undergoes an about 11-year cycle. HRV decreases associated with geomagnetic storms, which occur more frequently during solar maxima, may increase the cardiovascular disease risk of susceptible individuals. Such an outcome was witnessed in data on mortality from myocardial infarction in Minnesota, which show a 5% increased mortality during years of maximal solar activity (P = 0.023)^71^. Factors underlying the difference in response observed in space or subarctic areas will need further investigation based on concomitant longitudinal geomagnetic and biomedical monitoring in order to better understand any anti-aging benefits of the space environment, which could then serve to design countermeasures to prevent adverse vascular events on Earth.

Our previous investigation showed a graded response of HRV depending on the degree of geomagnetic activity (gauged by the geomagnetic index ap) in healthy young men living above the arctic circle^72,73^. Specifically, as compared to days when ap < 7, on days when 7 < ap < 20 or days when 20 < ap < 45, TF was decreased by 18.1% and 31.6%, ULF by 18.1% and 27.5%, and VLF by 12.9% and 28.6%, respectively. A graded decrease of HRV to geomagnetic activity suggests the existence of human magnetoreceptors. Phillips *et al*.^74^ proposed a light-dependent magnetoreception mechanism and established a link between magnetic field sensitivity and the visual system in eastern red-spotted newts. A magnetoreception mechanism may thus also exist in humans.

Cryptochromes are blue-light absorbing flavoproteins that in animals have an important function in the circadian clock^75–77^. Furthermore, it has been suggested that cryptochromes act as receptor molecules with magnetically sensitive radical pair reactions in the light-dependent magnetic compass sense of life on Earth^78–82^. Many animals, including birds, flies, bats, turtles, ants, mole rats, foxes, cows and deer, use the Earth’s magnetic field for orientation and navigation. Nießner *et al*.^83^ also localized *Cry1* in the retina of Canidae, Mustelidae, Ursidae and some Primates. While humans are not consciously aware of changes in magnetic field and while human magnetoreception has been rarely tested, yielding inconclusive results, a new study^84^ reports a strong and reproducible human brain response to geomagnetic stimulation, involving a drop in amplitude of EEG alpha oscillations (8–13 Hz) in association with horizontal rotations when the static vertical field is directed downwards. Such alpha event-related desynchronization has previously been linked to sensory and cognitive processing of external stimuli, including vision, auditory and somatosensory cues. Humans are also known to have two cryptochromes (CRY1 and CRY2), which act as clock proteins coordinating the circadian system. Foley *et al*.^85^ found that human cryptochrome is sensitive to blue light. Using a transgenic approach, they showed that human CRY2 can act as a magnetic sensor.

Mutant flies lacking the gene for cryptochrome did not respond to magnetic fields, but by transfecting them with two different cryptochrome genes from monarch butterflies they responded to magnetic fields under full-spectrum light^78,80^. These deletion and replacement experiments showed that the cryptochrome is an essential feature of the light-dependent magnetic sensing system in animals. These animals, however, were exposed to magnetic fields 8 to 10 times stronger than the geomagnetic field on Earth, and similar experiments cannot be conducted in humans, of course.

Our previous investigation showing HRV suppression on days of high geomagnetic disturbance^24^ found a decrease in HRV only when daylight alternated with darkness (above the arctic circle, there are days of complete light or darkness), suggesting a mechanism sensitive to the alternation of light (L) and darkness (D). The hypothesis of a light-dark-influenced magnetoreception was supported by cross-spectral analysis. Group-averaged coherence at frequencies coincident with the geomagnetic Pc6 pulsations (periods ranging from 10 minutes to 5 hours) differed among the three natural lighting conditions, the association being weaker during L/L or D/D than during D/L (P < 0.000001). A light-dark-influenced magnetoreception mechanism in humans may thus involve the Pc6 band of the magnetic field. Only the magnetoreception in HRV VLF-component was equal throughout the seasons, irrespective of the three natural lighting conditions, suggesting that biological signals involving VLF, which is vital for life, are set-up to be highly sensitive to geomagnetics.

A circadian stage-dependent response of HRV to magnetic activity in space was also found in the present study, the most sensitive response being observed during the daytime (12:00–17:00) and evening (18:00–23:00) (Table 3), when the intensity of illumination on board was around 700 lux. This result is in agreement with the possibility that humans have a light-dependent magnetoreception mechanism that remains functional in space.

Recently we reported that adaptation to microgravity occurs during long-duration spaceflight primarily according to two primary processes, one involving the dynamics of large-scale brain networks, initiated by the default mode network (DMN), and another coordinated by the circadian system^11^. The adaptation process proceeded even in the absence of consciousness. Herein, we also observed an acceleration of the DMN activity gauged by HRV (Table 2). Our results lead to the hypothesis that exposure to magnetic variation in space, via the retina, or the brain directly, may activate the dynamics of large-scale brain networks, including the DMN. The resulting HRV changes involved an anti-aging effect, enhanced after 6 months in space, together with an adaptation to the novel space environment.

The idea that space-magnetics may directly affect DMN activity, perhaps bringing about an anti-aging effect, is not surprising in view of recent documentation of transcranial magnetic stimulation (TMS) results now being introduced into clinical practice^86–88^. Alterations in DMN activity patterns strongly correlated with depression. In depressed people, disordered activity in the DMN correlates with negative rumination. Liston *et al*.^89^ reported that TMS seemed to create a shift in the relationship between two nodes in the DMN, the medial prefrontal cortex and the dorsolateral prefrontal cortex. The medial prefrontal cortex is involved in threat assessment and emotional decision making. The dorsolateral prefrontal cortex, which is where TMS stimulation takes place, seems to be involved in more thoughtful decision making, holding multiple ideas in mind and trying to understand before judging. This shift in functional brain activity could account for the mechanism of action of TMS. It should be noted, however, that magnetic variations in space, of the order of 54000 nT with a SD of 35 to 70 nT, are more than 10,000 times weaker than TMS (1–2 Tesla).

### Limitations

This is the first report investigating the effect of magnetic activity on humans’ HRV in space. Whereas it may seem that differences in geomagnetic indices were stronger during ISS01 than during ISS02, these differences may stem mostly from smaller SDs rather than from larger differences *per se*. Accordingly, it may no longer be surprising that some differences in HRV endpoints are more strongly expressed during ISS02 than during ISS01, a fact that may also be accounted for by the longer adaptation of astronauts to the space environment (see e.g., trajectories of LF/HF, TF, ULF and LF components, and LF-band).

The study was limited by the fact that geomagnetic measures in Tromso, Norway, were used instead of space weather measurements on the ISS. Magnetic activity during ISS01 and ISS02 did not reach levels found during magnetic storms, as it did in our subarctic study. Further studies are needed to assess the relative benefits and risks associated with the full spectrum of space weather conditions on the ISS.

The relatively short ECG records of only 48 hours from only 7 astronauts, monitored twice during a 6-month space mission is another limitation. These records were obtained during a span shorter than the solar activity cycle. Future work is needed to determine whether similar results can be obtained on additional astronauts, preferably based on longer ECG records, so that graded HRV responses to space weather can also be explored. Although larger and longer studies will also be needed to decide whether the physiological changes observed herein actually reflect an anti-aging effect, our results contributed novel information to better understand aging.

## Conclusion

Life on Earth is protected by the atmosphere and the magnetic field. The Earth’s magnetic field can affect humans’ life, especially HRV indices^1,19–24,71–73^. We showed that the magnetic field can affect and enhance HRV indices involved in longevity, notably during the daytime and evening, probably in association with an elevated activity of brain’s DMN with help from the circadian clock. Anti-aging HRV indices were significantly increased on days of higher magnetic activity. The difference between our results herein and those of our previous observation in a subarctic area may relate to a difference in extent of disturbance of the magnetic field between the two studies.

Spaceflight produces changes that induce a state of frailty akin to aging, such as muscle and bone atrophy, and balance and coordination problems, which can mostly be reversed upon re-adaptation to life on Earth. As proposed by Vernikos *et al*.^17,90^, the aged human may reflect a figure of life as a long-term adaptation for avoiding gravity effects on Earth in the course of aging. Our results provide a start on a catalog of measurable components of aging, and indicate what may be effective ways to slow down or even reverse the aging process.
